# Dissecting placental host-pathogen interactions: Rift Valley fever virus infection in early human trophoblast stem cells

**DOI:** 10.1016/j.isci.2026.115584

**Published:** 2026-04-03

**Authors:** Yong-Dae Gwon, Sandra Haider, Martin Knöfler, Matthew Bradley, Johan Henriksson, Magnus Evander

**Affiliations:** 1 Department of Clinical Microbiology, Umeå University, Umeå 901 85, Sweden; 2 Umeå Center for Microbial Research (UCMR), Umeå University, Umeå 901 87, Sweden; 3 Placental Development Group, Reproductive Biology Unit, Department of Obstetrics and Gynecology, Medical University of Vienna, Vienna, Austria; 4 Department of Molecular Biology, Umeå University, Umeå 901 85, Sweden; 5 IceLab, Umeå University, Umeå 901 87, Sweden

**Keywords:** pathogenic organism, stem cells research, transcriptomics

## Abstract

Rift Valley fever virus (RVFV) is a mosquito-borne Phlebovirus and zoonotic pathogen affecting maternal-fetal health. Vertical transmission is linked to miscarriage and severe fetal outcomes, but mechanisms of placental pathogenesis remain unclear. We used first-trimester human trophoblast stem cells (hTSCs) to model infection at the maternal-fetal interface. Immunofluorescence, qRT-PCR, western blotting, and single-cell transcriptomics showed that hTSCs are highly susceptible to RVFV. Strand-specific viral transcriptomics confirmed the ambisense S segment and revealed preferential transcription of the M and S segments over L. RVFV induced G1 arrest, impairing trophoblast proliferation and differentiation, and drove widespread transcriptional reprogramming, including strong interferon lambda 1 (*IFNL1*) but modest type I interferon responses, and dysregulation of inflammatory and preeclampsia-associated genes such as *RUNX1* and *TGFBRAP1*. Recombinant IFN-λ pretreatment reduced RVFV protein expression, highlighting hTSCs as a robust model and IFN-λ as a promising antiviral strategy.

## Highlights

• Single-cell landscape of RVFV in primary human trophoblast stem cells
• RVFV specifically blocks the G1-to-S cell cycle transition in trophoblasts
• Cytotrophoblasts mount a frontline defense by producing type III interferon
• RVFV triggers inflammation and preeclampsia-associated gene signatures

## Graphical abstract

**Figure:**
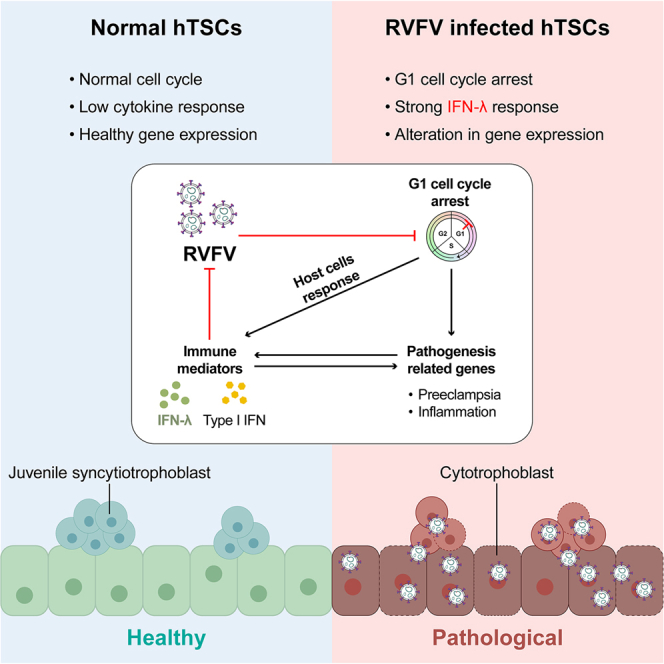

## Introduction

Rift Valley fever (RVF) is a vector-borne viral disease primarily affecting livestock, with occasional transmission to humans.^1^ The disease is caused by the Rift Valley fever virus (RVFV), a member of the *Phlebovirus* genus in the *Phenuiviridae* family.^2^ RVFV can be transmitted to humans through mosquito vectors (mainly *Aedes* and *Culex* species), and through direct contact with infected blood, aborted fetal tissue, or other contaminated biological materials during viremia.^3^ In humans, symptoms range from mild febrile illness to severe forms such as hemorrhagic fever, encephalitis, or ocular complications. Given its potential for zoonotic transmission and its impact on both public health and agriculture, RVF remains a significant concern in endemic regions^4^^,^^5^ and has the potential for global spread.^6^

Several viruses, such as the Zika virus, cytomegalovirus, and rubella virus, are well-documented causes of congenital disease through vertical transmission.^7^^,^^8^^,^^9^ The underlying mechanisms often involve maternal viremia, placental infection, and subsequent disruption of normal placental function.^10^ Similar mechanisms have been implicated in RVFV infection, where vertical transmission during pregnancy has been associated with a high risk of miscarriage and severe fetal outcomes in both humans and animals.^11^^,^^12^^,^^13^ In animals, studies suggest that maternal RVFV viremia facilitates vertical transmission, leading to placental damage and fetal demise. Histopathological analyses revealed extensive necrosis and hemorrhagic lesions in the placenta, indicating direct viral cytopathogenic effects.^12^^,^^13^ Pregnant women infected with RVFV may experience severe clinical manifestations, including hepatic dysfunction and hemorrhagic complications.^14^^,^^15^^,^^16^ Further research is needed to elucidate the precise mechanisms of RVFV-induced fetal loss and to develop targeted interventions for at-risk populations.

Infection during pregnancy could have significant implications for maternal and fetal health, since the immune system undergoes adaptations to maintain a delicate balance between protecting the mother from infection and preventing immune rejection of the fetus.^17^^,^^18^ Maternal immune responses, including innate and adaptive immunity, are activated during infection, but the immune system’s ability to respond may be modulated to avoid harming the fetus.^19^ Inflammatory cytokines and immune cells can affect placental function and fetal development, leading to complications such as preterm birth, fetal growth restriction, or miscarriage.^20^ Understanding the interplay between pathogen-induced immune responses and pregnancy-specific immune adaptations is crucial for developing effective therapeutic strategies.

The placenta plays a central role in this interplay, serving as both a physical and immunological interface between mother and fetus.^21^^,^^22^ In humans, the hemochorial placenta functions primarily through villous trophoblasts, which are essential for hormonal regulation, transport/exchange of nutrients and oxygen to the fetus, and secretion of cytokines and immunoregulatory factors.^23^^,^^24^ These functions not only support fetal growth and development but also actively modulate maternal immune responses, thereby influencing pregnancy outcomes under both physiological and pathological conditions.^21^^,^^22^^,^^23^^,^^24^

In a previous study, we used trophoblast cell lines to investigate the pathogenesis of RVFV during pregnancy; however, this model had inherent limitations because those cells are cancerous and/or immortalized.^25^ To obtain a better understanding of RVFV pathogenesis in a physiologically relevant context, more representative *in vitro* or *in vivo* models are needed. In this study, we utilized human trophoblast stem cells (hTSCs), which are defined by their capacity for long-term self-renewal and their multipotent potential to differentiate into all major placental lineages (26). Unlike pluripotent embryonic stem cells, hTSCs represent committed tissue-specific progenitors that can give rise to cytotrophoblast (CyT) subtypes, syncytiotrophoblast (STB), which form the multinucleated syncytial layer responsible for direct maternal-fetal exchange, and extravillous trophoblast (EvT) cells.^26^^,^^27^ Accordingly, hTSCs constitute a physiologically relevant model for investigating pathogen-induced infections during pregnancy.^28^

hTSCs possess the unique ability to self-renew and differentiate into STB and EvT, thereby closely recapitulating early placental development and barrier functions.^26^^,^^29^ This makes them particularly suitable for investigating how viruses interact with trophoblast cells and potentially breach the placental barrier.^29^ hTSC-based models offer a controlled *in vitro* system to study viral tropism, host-pathogen interactions, and immune responses within placental lineages. Previous studies using hTSCs have successfully elucidated mechanisms of infection and immunity in the context of congenital viruses such as the Zika virus, revealing entry pathways and cellular tropism.^30^^,^^31^ Applying this model to study RVFV infection during pregnancy allows for the identification of viral entry mechanisms, replication dynamics, and potential placental immune defenses. These insights are critical for understanding how RVFV compromises placental integrity, contributing to fetal infection and adverse pregnancy outcomes, and may inform the development of targeted therapeutic strategies to mitigate vertical transmission risks.

In this study, we employed immunofluorescence, quantitative real-time PCR (qRT-PCR), and single-cell transcriptome analysis using the PARSE Biosciences WT protocol to investigate RVFV transmission in hTSCs from first-trimester placentas and track the potential dynamic changes in the infected cells. Our data revealed the susceptibility of hTSCs to RVFV infection and identified genes potentially involved in the pathogenesis and immunomodulatory responses to RVFV. Notably, we highlighted type III interferon as a key component of the host defense mechanism and demonstrated the antiviral activity of interferon lambda (IFN-λ) against RVFV. Overall, our findings reveal the molecular interplay between immune response and RVFV pathogenesis in the first-trimester human placenta.

## Results

### First-trimester human trophoblast stem cells (hTSCs) are susceptible to RVFV infection

**Figure 1:**
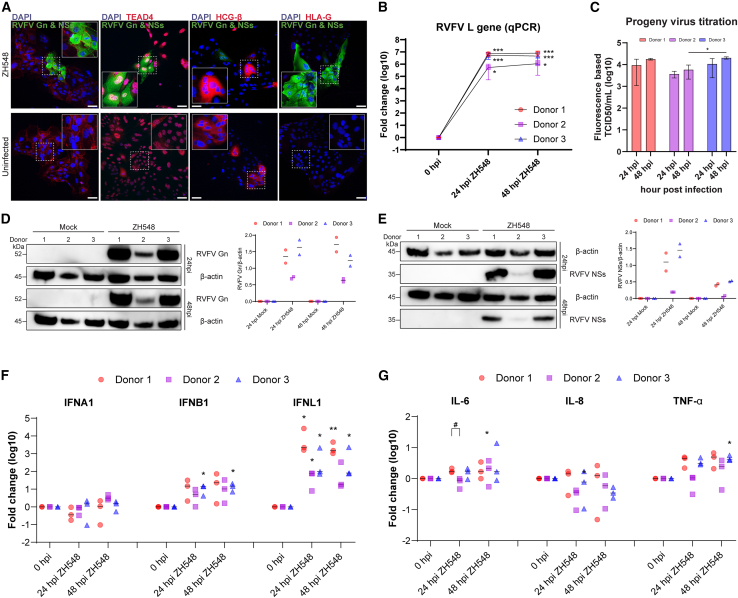
Characterization of RVFV infection and host response in human trophoblast stem cells (hTSCs) by immunofluorescence, qRT-PCR, and western blotting (A) Representative immunofluorescence microscopy of uninfected controls and RVFV strain ZH548-infected human trophoblast stem cells (hTSCs) from Donor 1 at 24 h post-infection (hpi). Cells were stained with lineage-specific markers: cytotrophoblasts (CyT) with E-Cadherin and TEAD4 (red), syncytiotrophoblasts (STB) with hCG-β (red), and extravillous trophoblasts (EvT) with HLA-G (red). RVFV infection was detected using an anti-RVFV Gn and NSs antibody (green), and nuclei were counterstained with DAPI (blue). Scale bars, 10 μm (main panels). (B) Viral replication kinetics of RVFV in hTSCs. Cells were infected with RVFV at an MOI of 1. Total RNA was extracted at 0, 24, and 48 hpi, and RVFV L gene expression was quantified by qRT-PCR. (C) Quantification of infectious progeny virus release. Viral titers in culture supernatants collected at 24 and 48 h post-infection (hpi) were determined using a fluorescence-based titration assay. The bar graph displays the log10-transformed infectious titers (fluorescence-based TCID50/mL) for three independent donors (donors 1–3). Error bars represent the standard error of the mean (SEM) of technical replicates. ∗*p* < 0.05 (two-way ANOVA), indicating a statistically significant increase in viral titers over time. Non-significant differences are not labeled. (D and E) Western blotting analysis of RVFV viral protein expression and quantification. Infection was performed as described in B. Lysates collected at 0, 24, and 48 hpi were analyzed for RVFV Gn and NSs proteins, normalized to β-actin. Each symbol represents a single data point from an independent experiment (*n* = 2). Statistical analysis was not performed due to the limited sample size. (F and G) Antiviral immune response (F) and inflammatory cytokine gene expression (G) in RVFV-infected hTSCs. The same synthesized cDNA from B was used to analyze the expression of type I (*IFNA1, IFNB1*) and type III (*IFNL1*) interferons (F), as well as inflammatory genes (*IL-6, IL-8*, and *TNF-α*) (G). General quantitative analysis (B, F, and G): For all qRT-PCR data, relative expression levels were calculated using the 2-ΔΔCt method, normalized to uninfected cells at 0 hpi. Data are stratified by donor to illustrate biological variability: donor 1 (red circles), donor 2 (purple squares), and donor 3 (blue triangles). Each symbol represents the mean of technical replicates, and horizontal lines indicate the mean of biological replicates. Statistical analyses were performed using two distinct approaches: (1) two-way ANOVA assessed donor variability, indicating no significant difference between donors across conditions, with the exception of *IL-6* at 24 hpi in F (#*p* < 0.05); (2) One-way ANOVA followed by Dunnett’s post hoc test assessed differences against the 0 hpi baseline for individual donors, indicated by asterisks (∗*p* < 0.05, ∗∗*p* < 0.01, and ∗∗∗*p* < 0.001).

To investigate host-pathogen interactions in a physiologically relevant model, primary hTSCs were isolated from first-trimester placental tissues obtained from three independent donors. Utilizing cells from all three biological replicates, we assessed the susceptibility of hTSCs to RVFV infection by examining the expression of trophoblast-specific lineage markers alongside two RVFV proteins, Gn and NSs, via immunofluorescence. As hTSCs represent a heterogeneous population that recapitulates key features of placental trophoblasts, this analysis enabled us to evaluate both their differentiation potential and infection status. The majority of cells expressed CyT markers, while smaller subsets showed positive expression of the STB marker, with no detectable expression of the EvT marker. Notably, CyT cells were permissive to RVFV infection, whereas susceptibility in STB cells remained inconclusive (Figure 1A).

To corroborate these findings, we performed viral replication kinetics assays based on the quantitative detection of the RVFV L gene (Figure 1B) and measured the release of infectious progeny virus (Figure 1C). Additionally, western blot analyses were conducted to evaluate the expression of Gn and NSs proteins (Figures 1D and 1E). Quantitative analysis revealed a significant time-dependent increase in viral RNA levels across all three donors, confirming productive replication. Consistent with this, western blot analysis demonstrated clear viral protein expression in infected hTSCs. Although some biological variability was observed in the magnitude of expression among donors, the susceptibility to RVFV was consistent across all biological replicates.

Previously, we found that RVFV induces a strong interferon lambda 1 (*IFNL1*) and inflammatory cytokine response in trophoblast cell lines (25). Therefore, we sought to determine whether a similar response occurs in primary hTSCs. Interestingly, hTSCs infected with the wild-type RVFV strain exhibited a significant upregulation of *IFNL1* mRNA expression across all donors. In contrast, type I interferons (*IFNA1* and *IFNB1*) showed only modest increases, with no significant induction observed compared to the baseline (Figure 1F). Regarding inflammatory cytokines, RVFV-infected hTSCs showed significant upregulation of *IL-6* RNA, particularly at 48 h post-infection (hpi), although donor-dependent variability was noted at earlier time points. In the case of *TNF-α*, increased expression was consistently detected in infected cells. In contrast, *IL-8* expression remained largely unchanged, with no significant differences observed compared to the uninfected control (Figure 1G). These findings suggest that *IFNL1* may be particularly responsive to RVFV infection in the early human placenta and may play a dominant role in regulating the immune response to pathogen exposure.

### Single-cell transcriptome analysis of human trophoblast stem cells infected by rift valley fever virus

**Figure 2:**
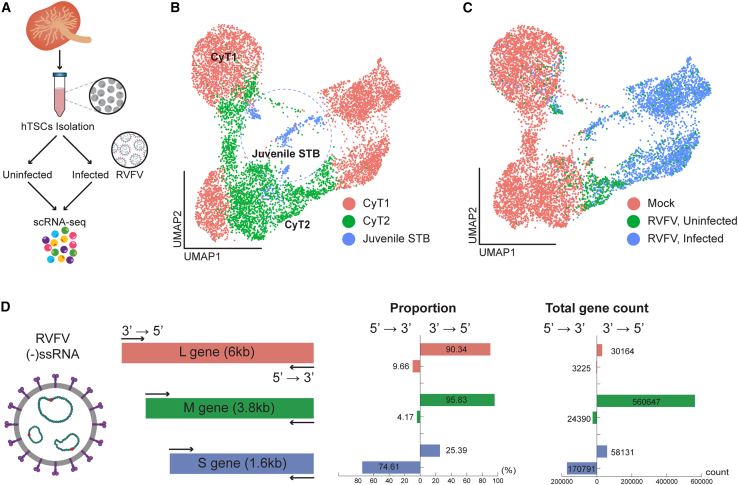
Single-cell RNA sequencing reveals cell-type distribution and RVFV infection dynamics in hTSCs (A) Schematic overview of the experimental workflow. Primary hTSCs were infected with wild-type RVFV (ZH548 strain) for 24 h, followed by single-cell RNA sequencing (scRNA-seq) to profile host-pathogen interactions. (B and C) UMAP visualization of the host transcriptomic landscape. (B) UMAP plot colored by cell type (CyT1, CyT2, and Juvenile STB), showing the distribution of trophoblast lineages within the infected and uninfected clusters. CyT, cytotrophoblast; Juvenile STB, juvenile syncytiotrophoblast. (C) Cells are colored by infection status, revealing three distinct populations: Mock (uninfected control), RVFV-uninfected (bystander cells exposed to the virus but lacking detectable viral RNA), and RVFV-infected (productively infected cells). Note that RVFV-infected cells form a spatially distinct cluster, indicating a profound reorganization of the transcriptome upon infection, whereas Mock and Bystander populations cluster closely together. (D) Strand-specific transcriptional activity of the RVFV genome. The transcriptional dynamics of the RVFV L, M, and S segments were analyzed to determine strand orientation and relative abundance.

To further investigate RVFV infection heterogeneity and its impact on placental cells at single-cell resolution, we exposed hTSCs to wild-type RVFV for 24 h and performed single-cell RNA sequencing (scRNA-seq) (Figure 2A). Three distinct clusters were annotated using established trophoblast markers, identifying cytotrophoblasts (CyT1 and CyT2) and juvenile syncytiotrophoblasts (juvenile STB) (Figure S2A). Given the importance of viral entry, we examined the expression of *LRP1*, a known host receptor for RVFV. We found *LRP1* to be broadly expressed across all identified clusters (Figure S2B).

To dissect the host response with greater granularity, we classified the cells into three distinct groups: uninfected controls (Mock), cells exposed to the virus but lacking detectable viral RNA (RVFV-uninfected/Bystander), and productively infected cells (RVFV-infected). Consistent with the ubiquitous expression of *LRP1*, viral RNA was detected across all trophoblast cell types, confirming that both CyT and STB lineages are susceptible to infection (Figures 2B and 2C). Notably, while the Mock and RVFV-uninfected populations clustered relatively closely, the RVFV-infected cells formed a spatially distinct cluster, indicating that productive infection induces a profound and complete reorganization of the host transcriptomic landscape (Figure 2B).

We then examined the transcriptional activity of RVFV’s three-segmented genome (S, M, and L) in a strand-specific manner to assess orientation-dependent expression patterns. As expected for a negative-sense single-stranded RNA virus, the transcription of the L and M segments predominantly occurred in the 3’ → 5′ direction, accounting for 90% and 96% of transcripts, respectively. Interestingly, the S segment exhibited a distinct pattern, with 74% of its transcription occurring in the 5’ → 3′ direction and only 25% in the 3’ → 5′ direction. This finding is consistent with the ambisense nature of the S segment, which encodes the NSs protein in the 5’ → 3′ orientation (Figure 2D).

In terms of overall transcriptional activity, we observed that the M and S segments exhibited 17.5-fold and 7-fold higher transcript counts, respectively, compared to the L segment. This suggests the differential regulation of viral RNA during infection (Figure 2D).

### Type III interferon signaling plays a critical role in defending against RVFV infection in hTSCs

**Figure 3:**
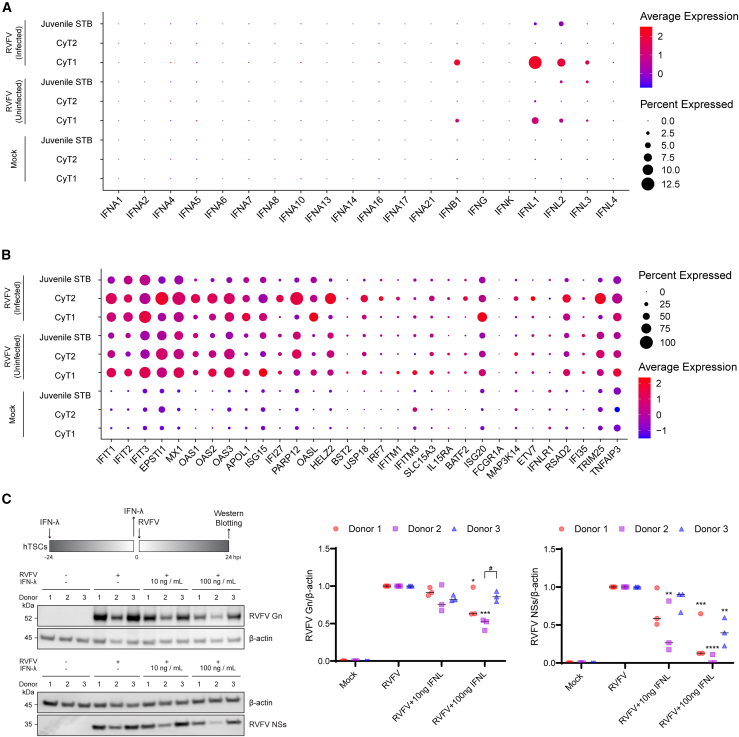
IFN-λ-mediated antiviral response against RVFV in hTSCs (A and B) Single-cell transcriptomic analysis of interferon and ISG expression. (A) Expression levels of type I (*IFNA1, IFNB1*) and type III (*IFNL1*) interferons. (B) Induction of key interferon-stimulated genes (ISGs). Data were derived from the scRNA-seq dataset described in Figure 2. Cells were stratified into three groups: Mock (uninfected control), RVFV-uninfected (bystander), and RVFV-infected, and expression values represent the average of three independent donors (*n* = 3) across trophoblast subtypes (CyT1, CyT2, and Juvenile STB). (C) Interferon lambda (IFN-λ) bioassay. hTSCs were pretreated with recombinant IFN-λ (10 ng/mL or 100 ng/mL) for 24 h prior to RVFV infection. (Left) Western blot analysis of RVFV Gn and NSs protein levels. β-actin was used as a loading control. Numbers 1–3 on the blot images correspond to the three individual donors. (Right) Densitometric quantification of Gn and NSs protein expression normalized to β-actin. Data are stratified by donor to illustrate biological variability: donor 1 (red circles), donor 2 (purple squares), and donor 3 (blue triangles). Each symbol represents the result of an independent experiment. Statistical analyses were performed using two distinct approaches: (1) two-way ANOVA assessed donor variability, indicating no significant difference between donors across most conditions, with the exception of Gn protein levels in the 100 ng/mL IFN-λ treatment group, where a significant difference was observed between Donor 2 and Donor 3 (#*p* < 0.05); (2) one-way ANOVA followed by Dunnett’s post hoc test was used to determine statistical significance against controls. Comparisons were made against the RVFV-infected untreated control group (∗*p* < 0.05, ∗∗*p* < 0.01, ∗∗∗*p* < 0.001, and ∗∗∗∗*p* < 0.0001).

Following RVFV infection in hTSCs, we monitored the regulation of interferon genes using our single-cell transcriptome dataset, stratifying cells into Mock, RVFV-uninfected (bystander), and RVFV-infected groups. While type I interferon genes (*IFNA1*) remained largely transcriptionally silent, *IFNB1* and type III interferon (*IFNL1*) were induced exclusively in the RVFV-infected population, with little to no upregulation observed in bystander cells. Notably, *IFNL1* exhibited the most pronounced upregulation compared to type I interferons, with higher expression levels observed primarily in cytotrophoblasts (CyT1) compared to juvenile STB (Figure 3A). Concomitantly, we observed a dynamic shift in the expression profile of interferon-stimulated genes (ISGs). These genes were strongly induced in the infected population, with expression patterns displaying cell type-dependent heterogeneity across the trophoblast lineage (Figure 3B).

To investigate the functional antiviral activity of IFN-λ against RVFV, hTSCs were pretreated with recombinant IFN-λ protein at concentrations of 10 ng/mL and 100 ng/mL for 24 h prior to infection. At 24 h post-infection (hpi), expression levels of the viral proteins Gn and NSs were evaluated. Notably, while untreated infected cells showed the highest viral protein expression, pretreatment with recombinant IFN-λ at 100 ng/mL inhibited the expression of both Gn and NSs proteins (Figure 3C). Although some donor-specific variability was noted in the magnitude of Gn suppression, the overall antiviral effect was significant, confirming that *IFNL1* upregulation serves as a critical defense mechanism in hTSCs.

### RVFV modulates the expression of both inflammatory and preeclampsia-associated genes in hTSCs

**Figure 4:**
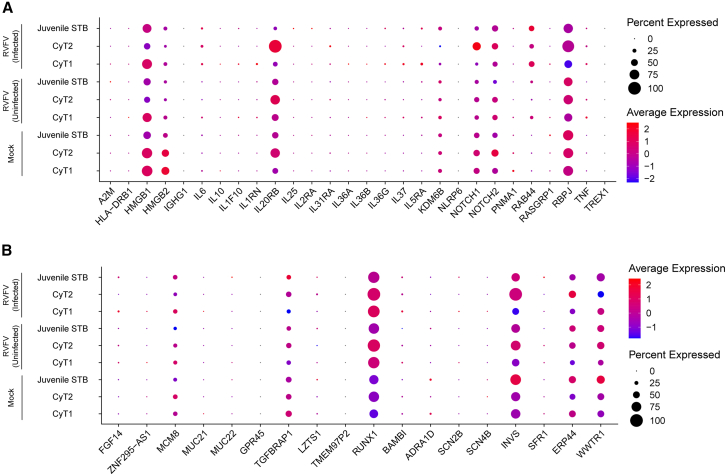
Transcriptional dysregulation of inflammatory and preeclampsia-associated genes in RVFV-infected hTSCs (A) Dot plot represents the expression levels of key inflammatory genes. (B) Dot plot represents the expression levels of preeclampsia-associated genes. Target genes were selected based on the GWAS Catalog annotation for preeclampsia (EFO_0000668). General quantitative analysis: Data were derived from the single-cell transcriptomes of hTSCs, where expression values represent the average of three independent donors (*n* = 3) across trophoblast subtypes (CyT1, CyT2, and Juvenile STB). Analyses were performed across three distinct populations: Mock (uninfected), RVFV-uninfected (bystander), and RVFV-infected, to assess infection-mediated transcriptional alterations. In the visualization, the size of each dot corresponds to the percentage of cells expressing the specific gene within each group, while the color gradient indicates the relative expression intensity (scaled average expression), highlighting transcriptional differences across the Mock, Bystander, and Infected conditions.

To dissect the molecular mechanisms underlying these adverse outcomes, we analyzed the expression of disease-associated gene sets across the Mock, RVFV-uninfected (bystander), and RVFV-infected populations (Figures 4A and 4B). First, given that inflammatory dysregulation at the maternal-fetal interface is a hallmark of recurrent spontaneous abortion (RSA),^32^ we examined a panel of inflammation-related genes. Dot plot analysis revealed a striking upregulation of the *HMGB1* gene, specifically within the RVFV-infected cluster (Figure 4A). As *HMGB1* functions as a potent damage-associated molecular pattern (DAMP), its specific induction in infected cells suggests that RVFV may trigger sterile inflammation. This creates a potential paracrine loop that recruits immune cells to the placental barrier, thereby exacerbating tissue damage.

Beyond the canonical inflammatory mediators, we observed critical alterations in genes governing epithelial defense and cellular trafficking. Notably, *IL20RB*, a receptor subunit for the tissue-protective cytokines *IL-19*, *IL-20*, and *IL-24*, was significantly dysregulated in the infected population. This transcriptional perturbation suggests a sensitized state to cytokine signaling, potentially aimed at maintaining barrier integrity. Concurrently, *RAB44*, an atypical Rab GTPase known to regulate granule exocytosis and lysosomal trafficking, was markedly induced. The upregulation of *RAB44* implies that RVFV infection triggers an enhancement of the cellular secretory machinery, likely to facilitate the rapid release of pro-inflammatory cytokines or viral factors from the trophoblasts.

Next, we assessed the expression of genes associated with preeclampsia susceptibility (based on GWAS Catalog EFO_0000668) to determine if viral infection mimics this pathological state (Figure 4B). We observed significant transcriptional perturbations in key developmental and signaling modules. Most notably, core components of the Notch signaling pathway (*NOTCH1*, *NOTCH2*, and *RBPJ*) were significantly dysregulated in the infected population compared to Mock and Bystander cells. Since Notch signaling is indispensable for trophoblast lineage specification and differentiation into EvT,^33^^,^^34^ this perturbation implies that RVFV infection actively impairs the developmental plasticity of the placenta. Such disruption hinders proper villous maturation, offering a molecular explanation for the placental insufficiency often observed in severe pregnancy complications, including preeclampsia.

### RVFV infection induces a disruption in cell cycle progression in hTSCs, leading to arrest at the G1 phase

**Figure 5:**
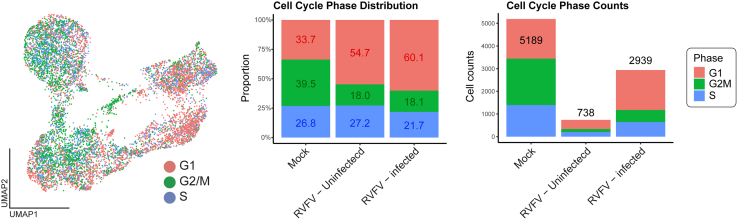
RVFV infection induces cell-cycle arrest in hTSCs (Left) UMAP visualization depicting the global cell cycle phase distribution of hTSCs. The plot integrates single-cell data from all experimental conditions, coloring cells according to their assigned cell cycle phase (G1, G2/M, or S) to illustrate the overall landscape of proliferation. (Right) Quantitative analysis of cell cycle phase distribution. The bar chart compares the percentage and absolute number of cells in each phase across three distinct populations: Mock (*n* = 5189), RVFV-uninfected (bystander) (*n* = 738), and RVFV-infected (*n* = 2939). Data are aggregated from three independent donors (*n* = 3). Note that the RVFV-infected population exhibits a distinct accumulation in the G1 phase, indicating viral-induced cell-cycle arrest compared to Mock and Bystander controls.

Given that RVFV is known to induce cell-cycle arrest in infected cells,^35^^,^^36^ we leveraged our single-cell resolution data to dissect the cell-cycle dynamics across Mock, RVFV-uninfected (bystander), and RVFV-infected populations. Visualizing the global cell cycle landscape revealed that while Mock and Bystander cells exhibited a typical distribution of proliferating cells, the RVFV-infected population displayed a distinct accumulation in the G1 phase (Figure 5). Quantitative analysis confirmed this observation: Approximately 60% of productively RVFV-infected cells were arrested in the G1 phase, a significant deviation compared to the Mock controls. This finding aligns with previous reports indicating that the ZH548 strain induces cell-cycle arrest specifically at the G0/G1 phase,^35^ indicating that RVFV infection actively interferes with cell cycle progression in human trophoblasts by blocking the G1-to-S phase transition.

## Discussion

Viral infections during pregnancy pose significant risks not only to maternal health but also to fetal development, often leading to adverse outcomes such as miscarriage, stillbirth, and congenital abnormalities.^37^ Among these, RVFV has the potential to cause spontaneous abortion in humans. Recent studies indicated that RVFV infection during pregnancy markedly increases the risk of miscarriage, likely through the direct infection of placental tissues, which triggers inflammation and structural damage.^12^^,^^38^ Despite these findings, the precise mechanisms by which RVFV induces pregnancy loss remain poorly understood.

Our study demonstrates that primary hTSCs derived from first-trimester placentas comprise multiple distinct trophoblast cell types and are highly permissive to RVFV infection. Although we observed donor-dependent variability in the magnitude of viral gene expression—most notably with one donor exhibiting intrinsically restricted susceptibility despite uniform culture conditions—the core pathological phenotypes were consistently conserved across all biological replicates. This observed variability likely reflects distinct genetic backgrounds or epigenetic landscapes inherent to primary tissues. Unlike genetically homogenous cell lines, this heterogeneity probably highlights a key advantage of the hTSC model, as it recapitulates the natural diversity of host responses found in the human population. This consistency in core pathology amid biological variability underscores the reliability of the hTSC model for studying RVFV pathogenesis.

Using single-cell transcriptomic profiling, we mapped early cellular responses to RVFV infection with high resolution. By stratifying cells into Mock, Bystander (RVFV-uninfected), and RVFV-infected populations, we revealed that productive infection drives a profound reorganization of the host transcriptome. Interestingly, the viral receptor *LRP1* was ubiquitously expressed across all clusters, supporting the broad susceptibility observed. Consistent with previous studies,^12^^,^^13^ this aligns with our finding that CyTs exhibited more transcriptional alterations compared to STBs. As CyTs serve as the proliferative progenitor pool for the placenta, their high permissiveness and distinct transcriptional response suggest that they may act as the primary amplification site for the virus before differentiation.^13^^,^^25^ The relatively limited response in STB cells likely reflects their differentiated status and specialized role in immune modulation at the maternal-fetal interface.^13^^,^^33^

Spontaneous abortions have been associated with dysregulated inflammation at the maternal-fetal interface.^32^ Our single-cell analysis highlighted that inflammatory changes were largely confined to the infected population. Specifically, the upregulation of *HMGB1*, a potent DAMP, suggests that RVFV infection triggers sterile inflammation.^34^^,^^39^ This could establish a paracrine loop that recruits immune cells to the placental barrier, potentially exacerbating tissue damage. Concurrently, key components of the Notch signaling pathway (*NOTCH1*, *NOTCH2*, and *RBPJ*) were significantly dysregulated in infected cells. Since Notch signaling is indispensable for trophoblast lineage specification and differentiation into EvTs,^40^^,^^41^ this perturbation implies that RVFV actively impairs the developmental plasticity of the placenta, hindering proper villous maturation.

We also observed a complex dysregulation of preeclampsia-associated genes. For instance, *RUNX1* was upregulated; altered *RUNX1* expression has been implicated in disrupted placental function and preeclampsia pathogenesis. Conversely, *TGFBRAP1*, involved in cell growth, was downregulated.^42^ These findings suggest that RVFV infection induces a “pathological reprogramming” of the trophoblast transcriptome that mimics the molecular signature of severe pregnancy complications.^43^

A critical finding of our study is the RVFV-induced cell-cycle arrest at the G1 phase in infected hTSCs.^35^^,^^36^ This phenotype, specific to the infected population, is consistent with the known effects of the viral nonstructural protein NSs. In the context of the first-trimester placenta, where rapid cell division is a prerequisite for the expansion of placental villi and successful implantation, such a blockade of the G1-to-S transition implies a critical disruption in trophoblast proliferation. Consequently, this specific induction of G1 arrest likely compromises the maintenance of the trophoblast progenitor pool. We propose that this physical blockade, combined with the transcriptomic dysregulation described above, synergistically contributes to the placental insufficiency and intrauterine growth restriction (IUGR) frequently observed in RVFV-associated cases.^11^

Our strand-specific transcriptomic analysis also revealed differential expression among the viral genome segments, with the M and S segments showing markedly higher transcriptional activity than the L segment.^44^^,^^45^^,^^46^ This imbalance likely reflects post-entry regulation aimed at maximizing the synthesis of structural proteins and immunomodulatory factors such as NSs.^46^^,^^47^ Notably, the specific abundance of S-segment transcripts aligns with recent studies showing that the S-segment antigenome is efficiently packaged into infectious particles. Additionally, the unique orientation-specific expression of the S segment supports its ambisense coding strategy https://paperpile.com/c/nFK1ea/rLxm.^48^

Collectively, our findings raise the question of whether adverse pregnancy outcomes are driven primarily by direct viral cytotoxicity or by transcriptomic dysregulation. We propose that these mechanisms contribute synergistically to placental pathology. The observed cell-cycle arrest and cytopathic effects may compromise the physical integrity of the placental barrier.^12^ Concurrently, the “subtle” yet significant alterations in the transcriptomic landscape—particularly the upregulation of inflammatory mediators and dysregulation of developmental genes—likely disrupt functional maternal-fetal crosstalk.^49^ This dual impact could precipitate vascular insufficiency or immune-mediated rejection, ultimately predisposing the pregnancy to miscarriage.^50^

Another key insight from our study was the identification of type III interferons, particularly IFN-λ, as central to the trophoblast antiviral response against RVFV infection. Our transcriptomic data revealed a predominant induction of *IFNL1* over type I interferons. This observation is particularly intriguing given that the viral non-structural protein NSs is a potent antagonist of host transcription.^51^ The robust expression of *IFNL1* suggests that in human trophoblasts, the kinetics of type III IFN induction may be sufficiently rapid to escape NS-mediated nuclear shutoff, or that this pathway is intrinsically less susceptible to viral antagonism. Consistent with this potential for escape, treatment with IFN-λ significantly reduced viral protein production, consistent with its role in strengthening epithelial barrier integrity.^52^^,^^53^ While IFN-λ has generally been associated with more localized and less systemic inflammation compared to type I interferons, reports also indicate that it can elicit pro-inflammatory signaling depending on the cellular context.^54^^,^^55^ Thus, our findings support a potential protective role for IFN-λ in the placenta, though further studies are required to dissect the full immunological consequences.

In a previous study, we employed trophoblast cell lines to investigate the pathogenesis of RVFV infection during pregnancy.^25^ However, several limitations emerged. First, the expression levels of viral receptors in these cell lines may be abnormally high or dysregulated compared to primary trophoblasts. Second, prolonged culture can lead to the accumulation of genetic mutations. Finally, while the placenta consists of diverse trophoblast subtypes, commonly used cell lines typically represent only a restricted subset of these populations. To overcome these constraints, the current study utilized primary hTSCs, which offer a physiologically relevant *in vitro* model capable of recapitulating the heterogeneity of the early placenta. Nevertheless, specific structural and lineage limitations must be acknowledged to contextualize our findings. As noted, our standard 2D maintenance conditions predominantly yield CyT and STBs, but do not support the extensive differentiation of EvTs. While 3D trophoblast organoids could theoretically provide a more complex architecture including EVTs, they characteristically exhibit an “inside-out” (apical-in) polarity, where the syncytial surface faces the enclosed central lumen.^56^ This orientation restricts the study of pathogen entry from the maternal circulation, as viruses introduced into the culture medium cannot readily access the apical surface without invasive microinjection.

Despite these inherent challenges, our 2D hTSC model effectively mimics the “villous epithelial barrier” configuration. This setup is particularly advantageous for modeling hematogenous transmission, allowing us to investigate the intrinsic susceptibility of the placental barrier to circulating RVFV (viremia). By overcoming the accessibility issues of organoids and the biological artifacts of cell lines, our findings demonstrate that hTSCs serve as an accessible platform for dissecting host-pathogen interactions. This model holds significant potential for broad applications in studying vertical transmission mechanisms and screening antiviral agents against emerging viral threats during pregnancy.

In conclusion, our study utilizing the biologically relevant hTSC model elucidates the cellular and molecular mechanisms underlying RVFV pathogenesis in the early human placenta. By identifying specific viral targets (e.g., G1 arrest and Notch signaling) and host defenses (IFN-λ), these insights advance our understanding of congenital infections and highlight potential therapeutic avenues to mitigate vertical transmission.

### Limitations of the study

While our study provides the first comprehensive single-cell transcriptomic landscape of RVFV infection in primary hTSCs, several limitations should be noted. First, although hTSCs serve as a highly physiologically relevant model that closely recapitulates early placental development, it remains an *in vitro* system. Consequently, it lacks the full anatomical complexity, mechanical forces, and systemic maternal immune interactions (such as decidual macrophages and natural killer cells) present in an intact human placenta. Second, our scRNA-seq analysis was performed at a single specific time point (24 h post-infection), which captures a critical snapshot of the host-pathogen interaction but may not fully delineate the continuous temporal dynamics of viral replication and immune resolution. Third, this study utilized the wild-type RVFV ZH548 strain; therefore, future studies incorporating various clinical isolates or attenuated strains are necessary to determine whether the observed transcriptomic perturbations and specific G1 cell-cycle arrest are universally conserved mechanisms or strain-dependent phenotypes. Finally, as our model primarily reflects first-trimester trophoblast biology, the findings may not fully extrapolate to RVFV infections occurring during the second or third trimesters, highlighting the need for further investigations using late-gestation placental models.

## Resource availability

### Lead contact

Requests for further information should be directed to the lead contact, Magnus Evander (magnus.evander@umu.se).

### Materials availability

This study did not generate new unique reagents.

### Data and code availability

• Data: The RNASeq data are available at the following link: https://www.ebi.ac.uk/biostudies/ArrayExpress/studies/E-MTAB-16352?query=E-MTAB-16352 (BioStudies: E-MTAB-16352).
• Code: All original code and results have been deposited at GitHub (https://github.com/henriksson-lab/rift-placenta2025).
• Other: Any additional information required to analyze the data reported in this article is available from the lead contact upon request.

## Acknowledgments

This work was supported by: the 10.13039/501100004359Swedish Research Council (VR; grant 2022-01055 to M.E., grant 2024-03952 and 2024-06085 to J.H.); the Medical Faculty, Umeå University to M.E.; 10.13039/501100002794Cancerfonden (grant #23 3102 Pj to J.H.); and the Austrian Science Funds (grant P31470 to M.K. and P36159 to S.H.). Computation was enabled by resources provided by (i) High Performance Computing Center North (HPC2N; Umeå University, Umeå, Sweden) and (ii) the National Academic Infrastructure for Supercomputing in Sweden (NAISS), partially funded by the 10.13039/501100004359Swedish Research Council through grant agreement no. 2022-06725. Experimental work was enabled by the μNisch Single-Cell Facility (Umeå University, Umeå, Sweden); the National Genomics Infrastructure (NGI), funded by VR and 10.13039/501100009252Science for Life Laboratory, Sweden.

Finally, we acknowledge the Biochemical Imaging Center at Umeå University and the National Microscopy Infrastructure, NMI (VR-RFI 2019-00217), for assistance in microscopy.

## Author contributions

Conceptualization: Y.D.G., J.H., and M.E.; methodology: Y.D.G., J.H., and M.B.; investigation: Y.D.G.; formal analysis: Y.D.G., S.H., M.K., M.B., J.H., and M.E.; resources: S.H., M.K., J.H., and M.E.; writing – original draft: Y.D.G., J.H., and M.E.; writing – review and editing: S.H., M.K., Y.D.G., M.B., J.H., and M.E.

## Declaration of interests

The authors declare no competing interests.

## STAR★Methods

### Key resources table

**Table:** 

REAGENT or RESOURCE	SOURCE	IDENTIFIER
**Antibodies**		

Rabbit monoclonal anti-E Cadherin	Cell Signaling Technology	Cat# 3195; RRID: AB_2291471
Rabbit polyclonal anti-TEAD4	Sigma	Cat# HPA056896; RRID: AB_2683268
Rabbit polyclonal anti-human chorionic gonadotropin beta (hCG-b)	Thermo Scientific	Cat# PA5-116189; RRID: AB_2900823
Rabbit monoclonal anti-human HLA Class 1 Antigen G (HLA-G)	Cell Signaling Technology	Cat# 79769S; RRID: AB_2799940
Mouse monoclonal anti-RVFV Gn protein	European Virus Archive - GLOBAL	Ref-SKU: 015A-03446
Mouse monoclonal anti-RVFV NSs protein	European Virus Archive - GLOBAL	Ref-SKU: 015A-03421
Rabbit polyclonal anti-β-actin	Santa Cruz Biotechnology	Cat# sc-130656; RRID: AB_2629465
Goat anti-Mouse IgG (H+L) Cross-Adsorbed Secondary Antibod, Alexa Fluor™ 488	Thermo Fisher Scientific / Thermo Scientific	Cat# A-11001; RRID: AB_2534069
Goat anti-Mouse IgG (H+L) Cross-Adsorbed Secondary Antibody, Alexa Fluor™ 555	Thermo Scientific	Cat# A-21422; RRID: AB_2535844
Goat anti-Mouse IgG (H+L) Secondary Antibody, HRP	Thermo Scientific	Cat# 31430; RRID: AB_228307
Goat anti-Rabbit IgG (H+L) Secondary Antibody, HRP	Thermo Scientific	Cat# 31460; RRID: AB_228341

**Bacterial and virus strains**		

Rift Valley fever virus (RVFV), wild-type strain ZH548	J Näslund et al.^57^	GenBank: DQ375403.1, DQ380206.1, DQ380151.1

**Biological samples**		

Human first-trimester placental tissue (3 donors)	Medical University of Vienna	Ethics approval number EK084/2009

**Chemicals, peptides, and recombinant proteins**		

Human fibronectin	Merck	Cat# 11051407001
Advanced DMEM/F12	Thermo Scientific	Cat# 12-634-010
HEPES (10 mM)	Thermo Scientific	Cat# 15-630-080
B-27 Supplement	Thermo Scientific	Cat# 17504044
Insulin-Transferrin-Selenium-Ethanolamine (ITS-X)	Thermo Scientific	Cat# 51500056
L-Glutamine (2 mM)	Gibco	Cat# 25030081
Recombinant human epidermal growth factor (rhEGF)	R&D Systems	Cat# 236-EG-01M
CHIR99021	Tocris	Cat# 4432
Rock Inhibitor (Y-27632)	Santa Cruz Biotechnology	Cat# sc-281642A
Dulbecco’s Modified Eagle’s Medium (DMEM)	Thermo Scientific	Cat# 12100061
4′,6-diamidino-2-phenylindole (DAPI)	Merck	Cat# 10236276001
Pierce LDS Sample Buffer	Thermo Scientific	Cat# NP0007
SuperSignal West Pico PLUS Chemiluminescent Substrate	Thermo Scientific	Cat# 34580
TrypLE	Thermo Scientific	Cat# 12605010
Deoxyribonuclease I (Dnase I) from bovine pancreas	Merch	Cat# DN25-100MG
RNase inhibitor	Promega	Cat# N2111
Recombinant IFN-λ protein	R&D Systems	Cat# 1598-IL
UltraPure™ 0.5M EDTA, pH 8.0	Thermo Scientific	Cat# 15575020

**Critical commercial assays**		

RNeasy Mini Kit	QIAGEN	Cat# 74106
High-Capacity cDNA Reverse Transcription Kit	Thermo Scientific	Cat# 374967
TaqMan Fast Advanced Master Mix	Thermo Scientific	Cat# 4444557
Eukaryotic 18S rRNA Endogenous Control VIC/MGB Assay	Thermo Scientific	Cat# 4319413E
TaqMan Assay: IFN-α1	Thermo Scientific	Assay ID: Hs00256882_s1
TaqMan Assay: IFN-β1	Thermo Scientific	Assay ID: Hs01077958_s1
TaqMan Assay: IFN-λ	Thermo Scientific	Assay ID: Hs00601677_g1
TaqMan Assay: IL-6	Thermo Scientific	Assay ID: Hs00985639_m1
TaqMan Assay: IL-8	Thermo Scientific	Assay ID: Hs00174103_m1
TaqMan Assay: TNF-α	Thermo Scientific	Assay ID: Hs00174128_m1
Evercode Cell Fixation Kit	PARSE Biosciences	Cat# ECFC3300
Evercode WT Mini v3 Kit	Parse Biosciences	Cat# ECWT3100

**Deposited data**		

Raw and processed single-cell RNA sequencing data	BioStudies	E-MTAB-16352
Custom R scripts for scRNA-seq analysis	GitHub	https://github.com/henriksson-lab/rift-placenta2025

**Experimental models: Cell lines**		

Vero B4 cells	Benjamin M et al.^58^	

**Software and algorithms**		

QuantStudio 5 Software v2.3	Thermo Scientific	https://www.thermofisher.com/se/en/home/technical-resources/software-downloads/quantstudio-3-5-real-time-pcr-systems.html
GraphPad Prism (Versions 11.0)	GraphPad Software	https://www.graphpad.com
ImageJ (Fiji, version 1.54p)	NIH	https://imagej.net/software/fiji/
split-pipe pipeline (v.1.3.1)	Parse Biosciences	https://www.parsebiosciences.com
STAR (v.2.7.11b)	Dobin A et al.^59^	https://github.com/alexdobin/STAR
Samtools	Danecek P et al.^60^	http://www.htslib.org/
Seurat R package (v.5.3.0)	Hat Y et al.^61^	https://satijalab.org/seurat/
R Statistical Software (Current version)	R Core Team	https://www.r-project.org/

**Other**		

Primers and probe targeting RVFV L-segment	Bird B H et al.^62^	

### Experimental model and study participant details

#### Tissue sampling

Human first-trimester (weeks 6-11) placental tissue from three donors was obtained from legal pregnancy terminations with the permission of the ethical committee of the Medical University of Vienna (EK084/2009 with annual renewal), requiring informed consent of donating women, with no compensation offered. The tissue was processed within 2 hours of collection.

All donors were female. Information on age, race, and ethnicity of the donors was not collected for this study. Donor characteristics were not used as variables in experimental design or statistical analysis. No randomisation or blinding was applied.

#### Human trophoblast stem cell (hTSC) isolation

As described previously,^56^ hTSC lines were established from isolated villous cytotrophoblasts of human single placentae. hTSC lines were maintained on human fibronectin (Sigma) pre-coated surface in advanced DMEM/F12 (Invitrogen) containing 10 mM HEPES (Gibco), 1×B-27 Supplement (Gibco), 1×Insulin-Transferrin-Selenium-Ethanolamine (ITS-X, Gibco), and 2 mM Glutamine (Gibco), 100 ng/mL recombinant human epidermal growth factor (rhEGF, R&D Systems), 3 μM CHIR99021 (Tocris), and 5 μM Rock Inhibitor (Y-27632, Santa Cruz) at 37°C with 5% CO_2_. All cells were routinely tested and confirmed to be negative for mycoplasma contamination.

#### Virus and cell line

The wild-type RVFV (ZH548 strain) was used in this study. All procedures involving the wild-type ZH548 strain were conducted under biosafety level 3 (BSL-3) conditions, in accordance with the regulations of the Swedish Work Environment Authority, permission 2020/017702. The cell line Vero B4 origin was from Benjamin et al.^58^

### Method details

#### Virus propagation

One day prior to infection, 2 × 10^6^ Vero B4 cells were seeded in T75 flasks (Sarstedt, Nümbrecht, Germany). Cells were then infected with RVFV at a multiplicity of infection (MOI) of 0.01 for 1 h and subsequently maintained in DMEM containing 1% FBS. Supernatants were harvested at 72 h post-infection, and viral titers were determined via plaque assay.

#### hTSC cell infection

hTSCs were infected with RVFV in serum-free advanced DMEM/F12 at an MOI of 1 for 1 hour. The inoculum was then removed, and fresh hTSC culture medium was added.

#### Immunofluorescence detection of trophoblast markers and RVFV viral proteins

Twenty thousand hTSCs were seeded on human fibronectin-coated 8-well chamber slides (Sarstedt) a day before infection. Infection was performed as described above. After 24 hours, cells were fixed with 4% paraformaldehyde for 30 minutes at room temperature. To enable antibody penetration, cells were permeabilized with 0.25% Triton-X in PBS for 5 minutes and washed thrice with PBS. The primary antibodies used were rabbit anti-E Cadherin (#3195, Cell Signaling Technology), rabbit anti-TEAD4 (HPA056896, Sigma), rabbit anti-human chorionic gonadotropin beta (hCG-b, PA5-116189, Thermo Scientific), and rabbit anti-human HLA Class 1 Antigen G (HLA-G, #79769S, Cell Signaling Technology). Mouse anti-RVFV Gn- and NSs protein antibodies (European Virus Archive - GLOBAL) were also applied to trace RVFV infection. Secondary antibodies, anti-mouse Alexa 488 and anti-mouse Alexa 555 (Thermo Scientific), were used for detection, and 300 nM 4′,6-diamidino-2-phenylindole (DAPI) was used for counterstaining the nucleus. Slides were examined with a Zeiss 710 confocal microscope (Carl Zeiss, Oberkochen, Germany).

#### Total cellular RNA isolation and RVFV RNA quantification

For total RNA isolation, we employed the same method as described in our previous work.^25^ Briefly, RVFV-infected hTSCs were washed with PBS, and total RNA was extracted using the RNeasy Mini Kit (QIAGEN, Hilden, Germany) according to the manufacturer’s instructions. RNA yield and purity were assessed using a NanoDrop spectrophotometer (Thermo Scientific). Complementary DNA (cDNA) was synthesized from the purified total RNA using the High-Capacity cDNA Reverse Transcription Kit (Thermo Scientific).

RVFV RNA was detected using a previously described primer and TaqMan probe targeting the L-segment, which encodes the RNA-dependent RNA polymerase, in conjunction with the TaqMan Fast Advanced Master Mix (Thermo Scientific) and the QuantStudio 5 Real-Time PCR System (Thermo Scientific).^62^^,^^63^ For normalization, Ct values of the endogenous control gene *18S rRNA* were obtained using the Eukaryotic *18S rRNA* Endogenous Control VIC/MGB Assay (Cat. No. 4319413E, Thermo Scientific).

#### Quantification of infectious progeny virus (viral titration)

To quantify the release of infectious progeny virus, supernatants collected from RVFV-infected hTSCs were analyzed using an immunofluorescence-based titration assay. Vero B4 cells were seeded at a density of 15000 cells/well in 96-well plates (Greiner CELLSTAR®, Greiner Bio-One, Kremsmünster, Austria) one day prior to infection. Infectious supernatants were serially diluted (4-fold) in serum-free Dulbecco’s Modified Eagle Medium (DMEM) supplemented with 0.2% penicillin/streptomycin and transferred to the Vero B4 monolayers. Following a 1-hour adsorption period at 37°C, the inocula were removed and replaced with maintenance medium (DMEM supplemented with 2% FBS).

At 48 hours post-infection (hpi), cells were fixed with 4% formaldehyde for 30 minutes. The plates were washed with phosphate-buffered saline (PBS, pH 7.4), permeabilized with 0.5% Triton X-100 in PBS containing 20 mM glycine for 5 minutes at room temperature (RT), and subsequently blocked with PBS containing 2% bovine serum albumin (BSA) for 30 minutes at RT. For immunodetection, cells were incubated overnight at 4°C with a mouse monoclonal antibody against the RVFV Gn protein (1:1000 dilution in blocking buffer). Following washing, cells were incubated for 30 minutes with a secondary Goat anti-Mouse IgG conjugated to Alexa Fluor 488 (1:2500; Thermo Fisher Scientific, Waltham, MA, USA) and DAPI (0.1 μg/mL) for nuclear staining.

#### Cytokine mRNA expression analysis by qRT-PCR

The same TaqMan Fast Advanced Master Mix, QuantStudio 5 Real-Time PCR System, and normalization method described above were used for quantifying cytokine mRNA expression by qRT-PCR.^25^ Each reaction was performed in a 20 μL volume using a 1:10 dilution of cDNA synthesized previously and run for 40 cycles under the default thermal cycling conditions recommended for the TaqMan Fast Advanced Master Mix (Thermo Scientific). TaqMan FAM/MGB probe assays (Thermo Scientific) were employed for detection. mRNA expression levels were assessed for the following cytokines using the corresponding TaqMan Assay IDs: *IFNA1* (Hs00256882_s1), *IFNB1* (Hs01077958_s1), *IFNL1* (Hs00601677_g1), *IL6* (Hs00985639_m1), *IL8* (Hs00174103_m1), and *TNFA* (Hs00174128_m1).

#### RVFV viral protein detection by western blotting

Human trophoblast stem cells (hTSCs) were seeded at a density of 250,000 cells per well onto human fibronectin-coated 12-well plates one day prior to infection. Cells were washed and infected with RVFV, and cells were collected at 24 and 48 hours post-infection. For protein extraction, cells were lysed on ice for 30 minutes in a buffer containing 50 mM Tris (pH 8.0), 150 mM NaCl, 1% Triton X-100, and protease inhibitors (Roche). Lysates were centrifuged at 4°C, and supernatants were mixed with Pierce LDS Sample Buffer (Thermo Scientific), then heated at 70°C for 10 minutes.

Proteins were separated by SDS-PAGE, transferred to PVDF membranes (Bio-Rad), and blocked with 5% non-fat dry milk in Tris-buffered saline containing 0.1% Tween-20 (TBS-T) for 1 hour at room temperature. Membranes were incubated overnight at 4°C with primary antibodies diluted in 5% BSA/TBS-T: mouse anti-RVFV Gn and NSs (1:1000) and rabbit anti-β-actin (1:5000; Santa Cruz Biotechnology). After washing, membranes were incubated for 1 hour at room temperature with horseradish peroxidase-conjugated anti-mouse and anti-rabbit secondary antibodies (Thermo Scientific, 1:5000). Signal detection was performed using SuperSignal West Pico PLUS Chemiluminescent Substrate (Thermo Scientific) and imaged with the Amersham Imager 680 (Cytiva). Band intensities were quantified using the GelAnalyzer plugin in ImageJ (Fiji, version 1.54p).^64^^,^^65^

#### IFN-λ treatment and RVFV infection

One day prior to IFN-λ treatment, 250,000 hTSCs were seeded onto human fibronectin-coated 12-well plates. Cells were then treated with IFN-λ at two concentrations (10 ng/mL and 100 ng/mL) in serum-free advanced DMEM/F12 medium (R&D Systems) for 24 hours. Cells were washed and infected with RVFV, and cells were collected at 24 hours post-infection. Protein extraction and Western blotting were performed as described above.

#### Single-cell library preparation and sequencing

At 24 hours post-infection, single-cell preparation was performed. Briefly, cells were rinsed with PBS and treated with a dissociation buffer containing TrypLE (Invitrogen), DNase (25 μg/mL, Roche), EDTA (0.5 mM, Invitrogen), and RNase inhibitor (1 U/mL, Thermo Scientific) for 10 minutes. Dissociated cells were collected by centrifugation at 400 × g for 5 minutes at 4°C. Subsequently, hTSCs were fixed using the Evercode Cell Fixation Kit (PARSE Biosciences) and passed through a 30 μm filter (pluriSelect). Fixed cells were then processed, and single-cell libraries were prepared using the Evercode WT Mini v3 protocol (Parse Biosciences). Pooled libraries were sequenced on an Illumina NovaSeq X using a shared 1.5B flow cell (970 million reads), with a read configuration of R1: 128 cycles and R2: 177 cycles.

### Quantification and statistical analysis

All statistical details of the experiments, including the exact value of “n” (representing the number of independent biological donors or independent experiments), definition of center, and dispersion measures (e.g., mean ± SD or SEM), can be found in the respective figure legends.

#### qRT-PCR analysis

Data analysis was performed using QuantStudio 5 Software v2.3 (Thermo Scientific). Relative gene expression levels were calculated using the ΔΔCt method, with normalization to uninfected cells at time point 0 or corresponding controls.

#### Viral titration

Fluorescence intensity was quantified using a Cytation 5 Cell Imaging Multi-Mode Reader (BioTek). The reciprocal half-maximal infectious dilution (ID50) was determined using a 4-parameter nonlinear regression analysis in GraphPad Prism 8.0 (GraphPad Software, San Diego, CA, USA).

#### Statistical analysis

All statistical analyses were performed using GraphPad Prism software (Version 7.0). Data are presented as mean ± standard deviation (SD) or mean ± standard error of the mean (SEM) as indicated in figure legends. To rigorously distinguish between biological variability and treatment effects, two distinct statistical approaches were employed: 1. Assessment of Donor Variability: A Two-way Analysis of Variance (ANOVA) was utilized to evaluate potential differences attributable to biological donors (n=3) across experimental conditions; 2. Assessment of Treatment Effects: To determine statistical significance between experimental groups (e.g., Mock vs. Infected, or Untreated vs. IFN-treated), a One-way ANOVA followed by Dunnett’s or Tukey's post hoc test was performed. Statistical significance was defined as ∗p < 0.05.

#### Analysis of single-cell RNA-seq data

The split-pipe v.1.3.1 pipeline (Parse Biosciences), STAR 2.7.11b^59^ was used to align FASTQ files to a combined reference genome (Homo_sapiens. GRCh38.112; Genbank accession number for RVFV genome: DQ375406.1, DQ380200.1, DQ380149.1). To analyze RVFV strand-specific transcriptional activity, Bam files were subset to relevant viral reads using the grep command. Any lines that contained a match to a viral segment (using nucleotide sequences DQ375406.1, DQ380200.1, DQ380149.1) was written to a new file. Viral read data from these files were then read into R and reads per cell were counted. In order to assign strandedness (either forward or reverse) to each read, SAM flags in the previously created viral BAM files were read using Samtools.^66^ Any reads with a flag value of 0 were labelled as a forward read, and any reads with a SAM flag of 16 were labelled a reverse read.

Single-cell analysis followed a standard workflow using Seurat v.5.3.0.^67^ In short, count data was loaded and filtered (<6k detected features, <50k UMIs, <20% mitochondrial reads). 3000 variable features were extracted. Cell cycle phase was inferred using the built-in cc.genes list. A UMAP was produced from the first 30 PCA components. Clusters were called using FindClusters (resolution 0.4) and identified using marker genes.

1. Stratification of Infection Status: To dissect host-pathogen interactions at single-cell resolution, cells were stratified into three distinct biological categories based on the detection of viral M segment transcripts. The classification threshold was determined empirically by examining the distribution of background reads in the uninfected control sample, rather than by an arbitrary statistical cutoff. Based on this qualitative assessment of background noise, a strict cutoff of 1.3 (log10 expression level) was established to distinguish genuine viral replication from technical noise. Mock: Cells from the uninfected control samples. Notably, a negligible number of cells in the Mock group exhibiting background signal above this threshold (n=16) were excluded from downstream analysis to ensure the purity of the negative control. RVFV-infected: Cells within the viral-exposed samples displaying transcript levels above the 1.3 cutoff, indicating active viral replication. RVFV-uninfected (Bystander): Cells within the viral-exposed samples but with transcript levels below the 1.3 cutoff, representing the bystander population that was exposed to the viral environment but did not sustain high-level viral transcription (Figure S1).
2. Cell Cycle Scoring: Cell cycle phases (G1, G2/M, S) were assigned to each individual cell using the “CellCycleScoring” function in Seurat. This algorithm calculates a score based on the aggregate expression of canonical cell cycle markers^68^ and classifies cells into the phase with the highest score.
3. Functional Gene Set Analysis: To investigate pregnancy-specific pathologies, a curated list of preeclampsia-associated genes was retrieved from the GWAS Catalog (Trait ID: EFO_0000668). Inflammation-related genes were selected based on Molecular Signatures Database (MSigDB) Hallmark Inflammatory Response gene set (M5932) and transcriptional signatures of the early human placenta defined by Vento-Tormo et al.^69^^,^^70^ Differential expression patterns were visualized using Dot Plots to compare transcriptional perturbations across Mock, Bystander, and Infected groups.

## Supplemental information

Document S1. Figures S1 and S2

Document S2. Original images _spl_Western blotting_spl_260327
