# Supplementary material for: Dissecting placental host-pathogen interactions: Rift Valley fever virus infection in early human trophoblast stem cells
Source: iScience. 2026 Apr 3;29(5):115584. doi: 10.1016/j.isci.2026.115584 (PMC13098504; doi:10.1016/j.isci.2026.115584)
Supplement: Document S1. Figures S1 and S2 [file mmc1.pdf]

iScience, Volume *29*

Supplemental information

Dissecting placental host-pathogen interactions: Rift Valley fever virus infection in early human trophoblast stem cells

Yong-Dae Gwon, Sandra Haider, Martin Knöfler, Matthew Bradley, Johan Henriksson, and Magnus Evander

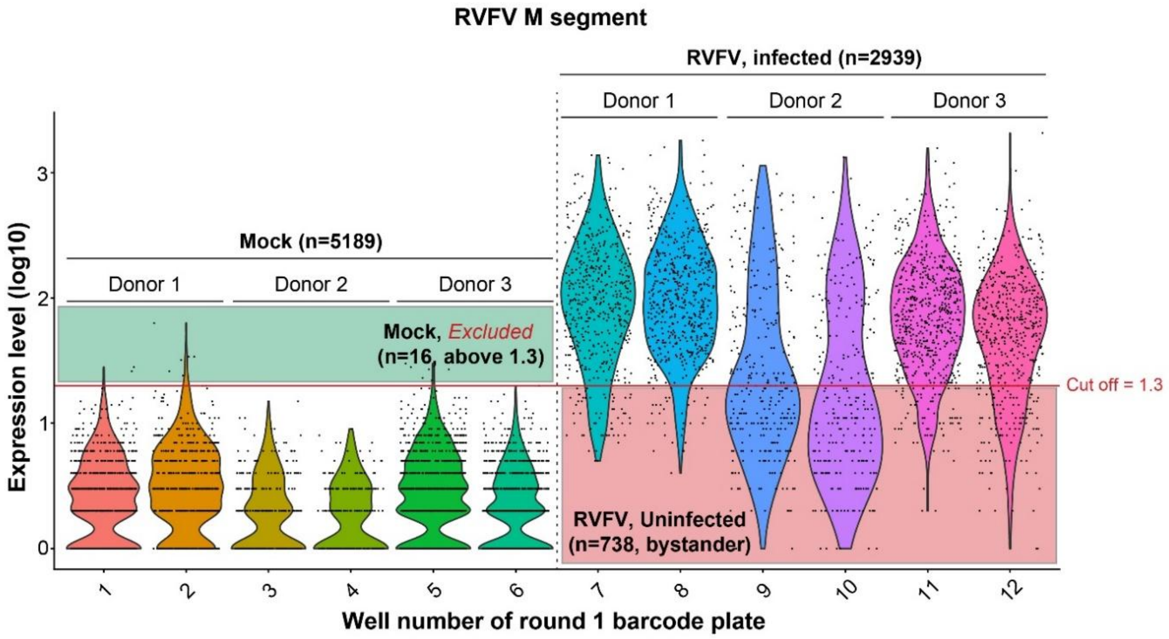

Figure S1. Stratification of single cells based on viral transcript levels. Violin plots display the distribution of RVFV M segment expression (log10-transformed) across individual cells, separated by donor and experimental condition. The red horizontal line indicates the empirically determined cutoff value of 1.3, selected based on the distribution of background noise in the uninfected control (Mock) group. Based on this threshold, cells were stratified into three categories: (1) Mock: Cells from uninfected wells. Outliers exhibiting background signal above the cutoff (n=16, green shaded region) were excluded as technical noise. (2) Bystander (RVFV-uninfected): Cells from the infected condition falling below the cutoff (n=738, pink shaded region), representing the exposed but uninfected population. (3) RVFV-infected: Cells from the infected condition exhibiting expression levels above 1.3 (n=2939), indicating productive infection.

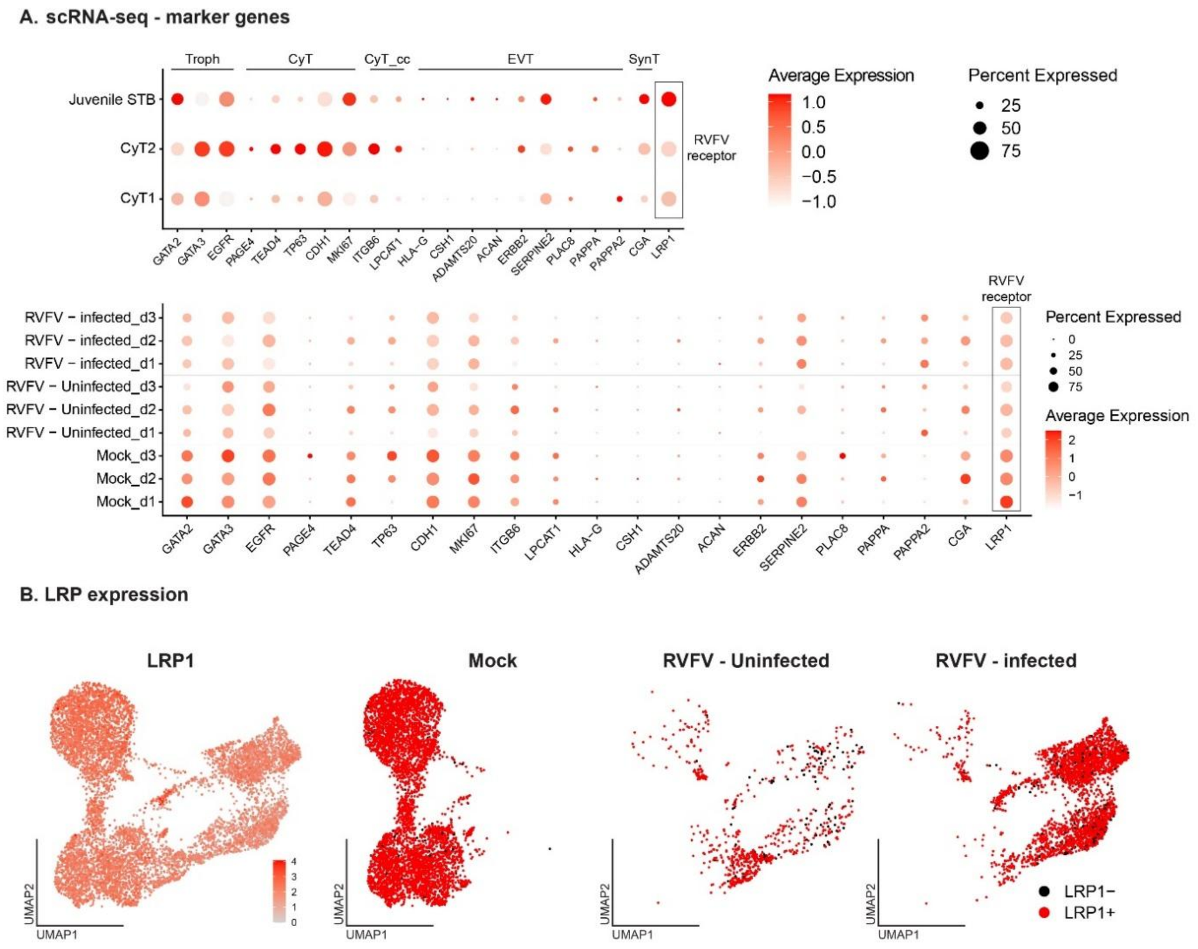

Figure S2. Expression of trophoblast lineage markers and the RVFV entry receptor LRP1. Figure S2. Expression of trophoblast lineage markers and the RVFV entry receptor LRP1. (A) Dot plots visualizing the expression of canonical trophoblast lineage markers (e.g., TEAD4, TP63, CGB) and the putative RVFV entry receptor, LRP1. The upper panel displays gene expression across the defined cell clusters (CyT1, CyT2, Juvenile STB), grouped by their differentiation status, representing the aggregated profile derived from three independent donors. In contrast, the lower panel stratifies expression by experimental condition (Mock, RVFV-uninfected, RVFV-infected) and individual donors (d1–d3). The size of each dot represents the percentage of cells expressing the gene, while the color intensity indicates the average scaled expression level. The rectangular outline highlights LRP1 as the viral receptor. (B) Feature plots projecting LRP1 expression onto the UMAP embedding. The leftmost plot illustrates the gradient of LRP1 expression across the entire dataset. The subsequent panels display the distribution of LRP1-positive cells (red) across the three biological conditions: Mock, RVFV-uninfected (Bystander), and RVFV-infected. Note the ubiquitous expression of LRP1 across the trophoblast clusters regardless of infection status.
