# Supplementary material for: Dissecting placental host-pathogen interactions: Rift Valley fever virus infection in early human trophoblast stem cells
Source: iScience. 2026 Apr 3;29(5):115584. doi: 10.1016/j.isci.2026.115584 (PMC13098504; doi:10.1016/j.isci.2026.115584)

Original Western Blot
Figure 1 D-E, Experiment 1

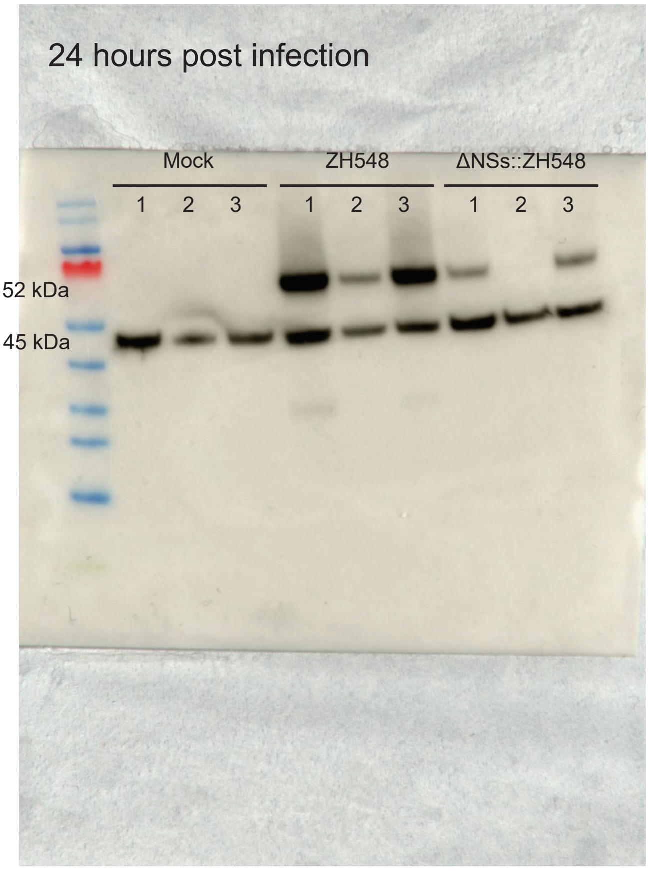

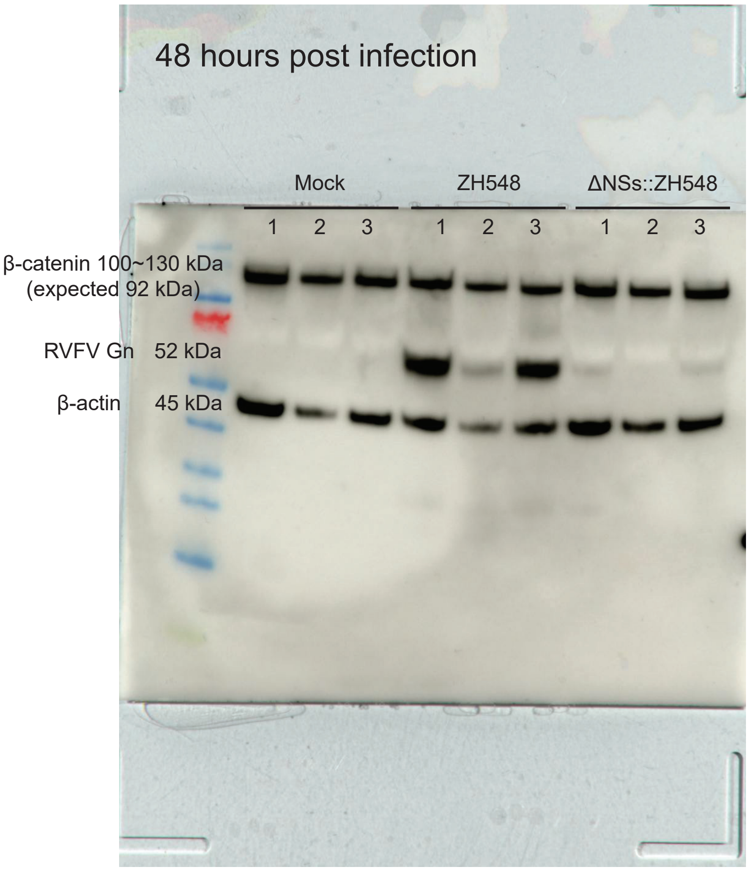

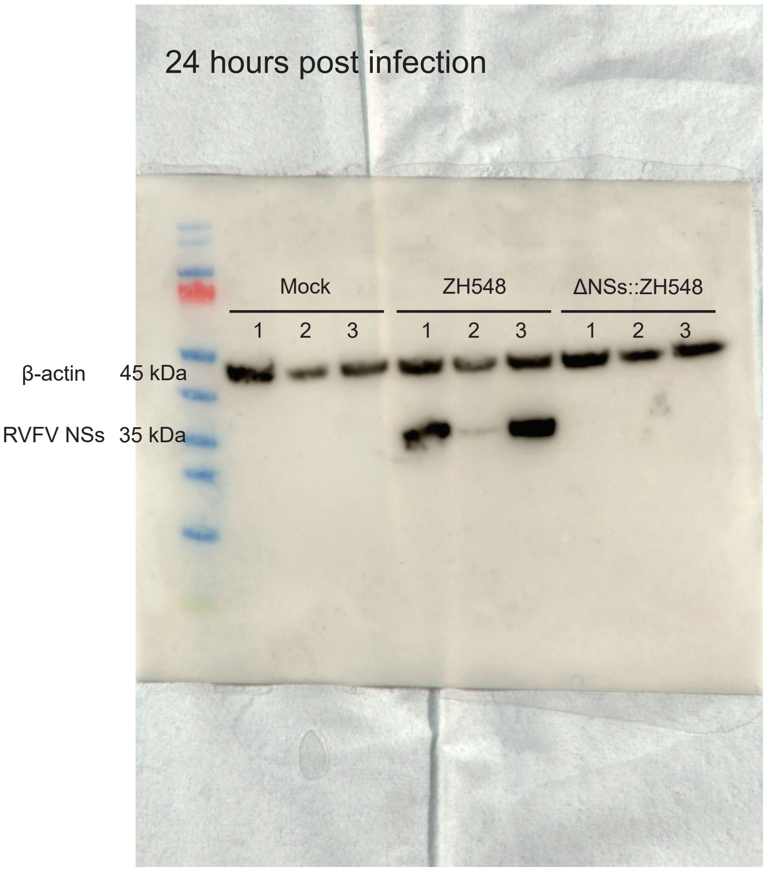

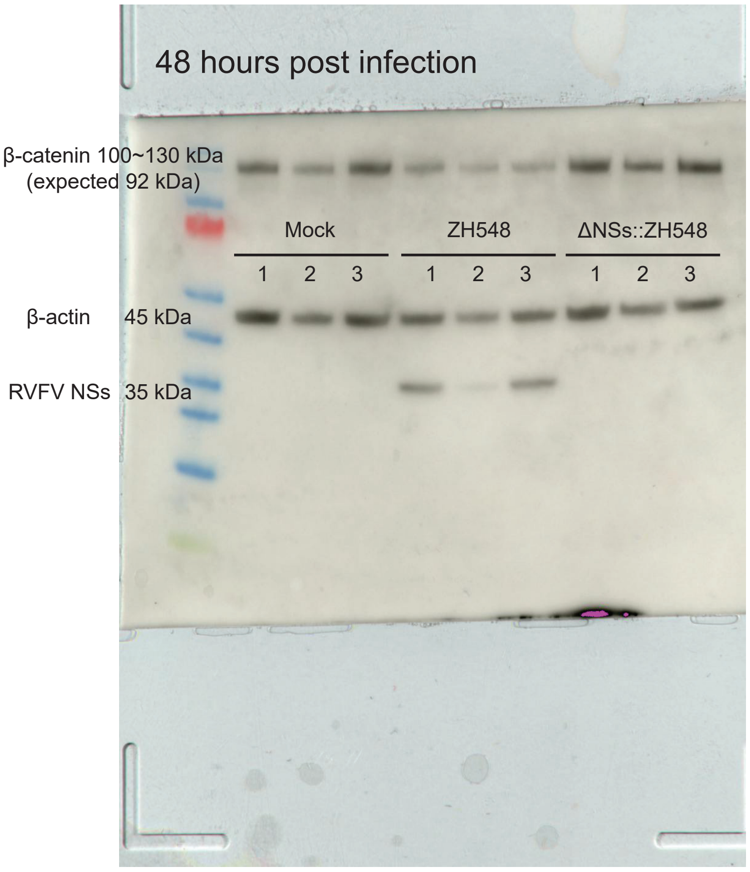

Original Western Blot
Figure 1 D-E, Experiment 2

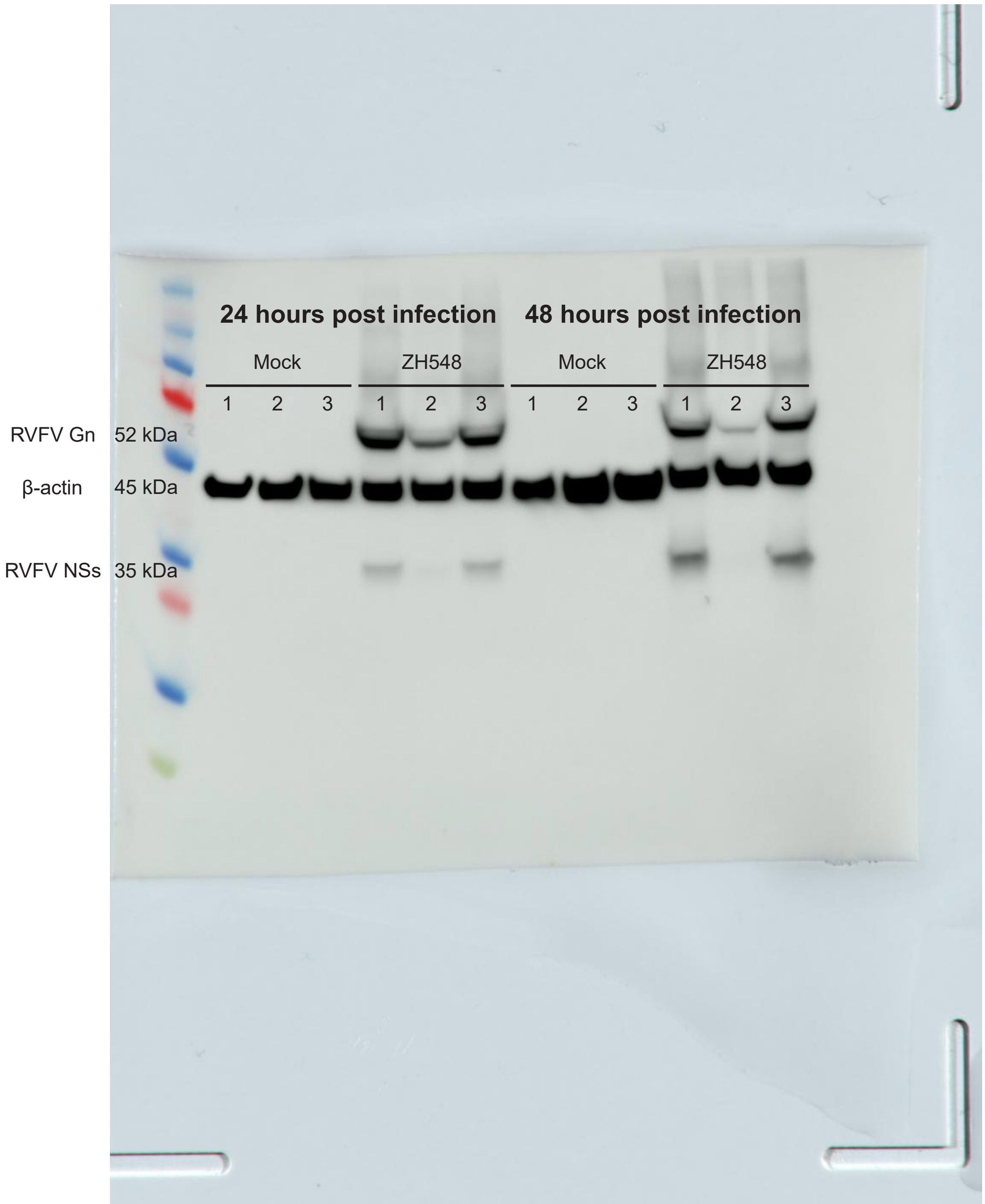

Original Western Blot
Figure 3 C, Experiment 1

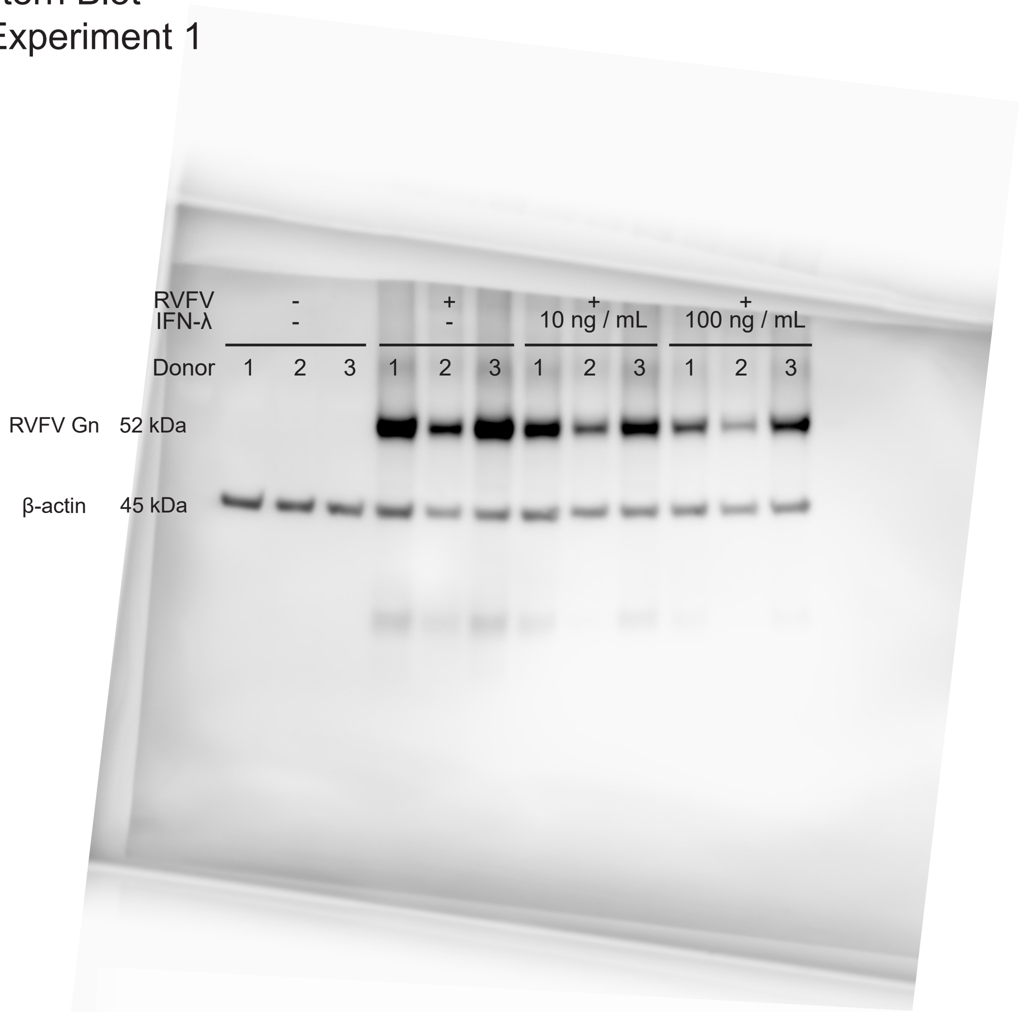

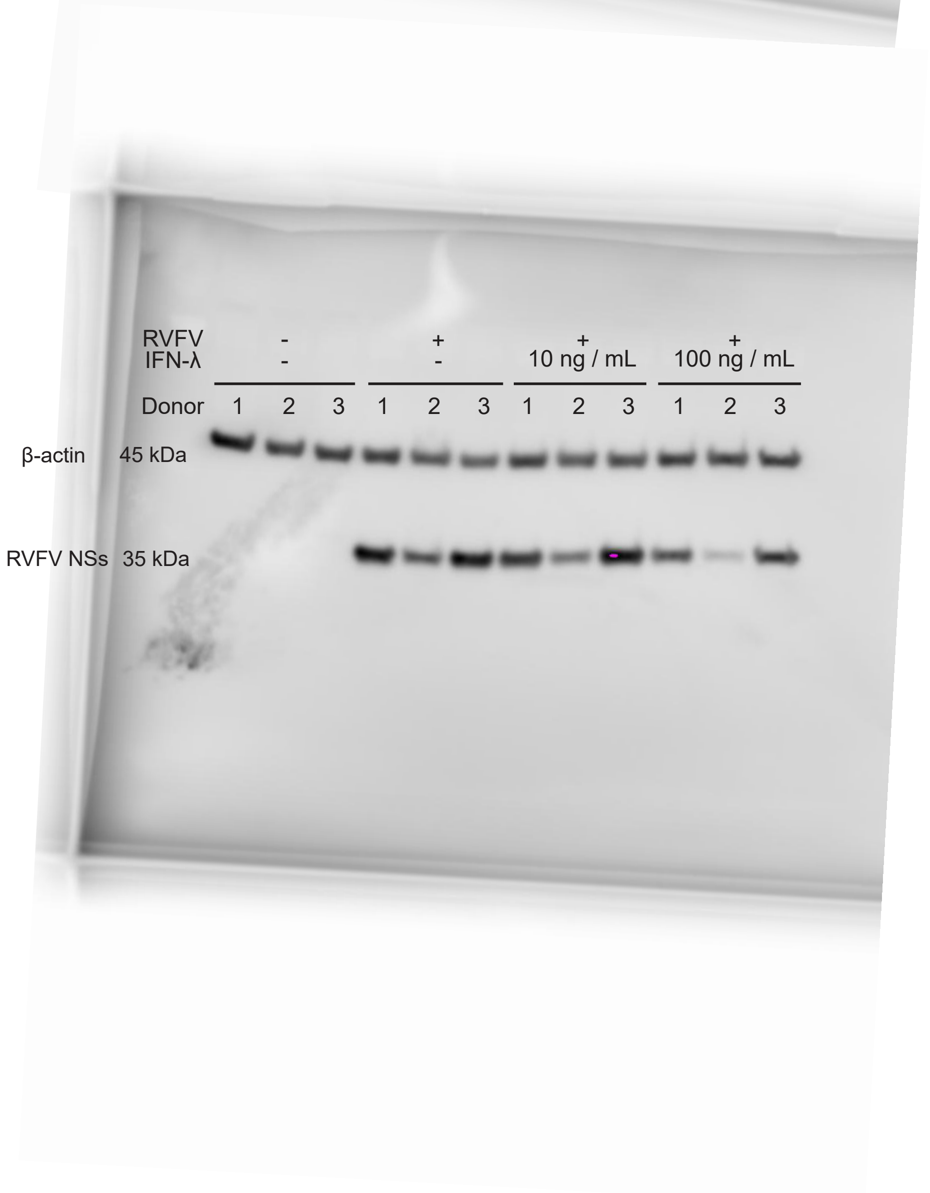

Original Western Blot
Figure 3 C, Experiment 2

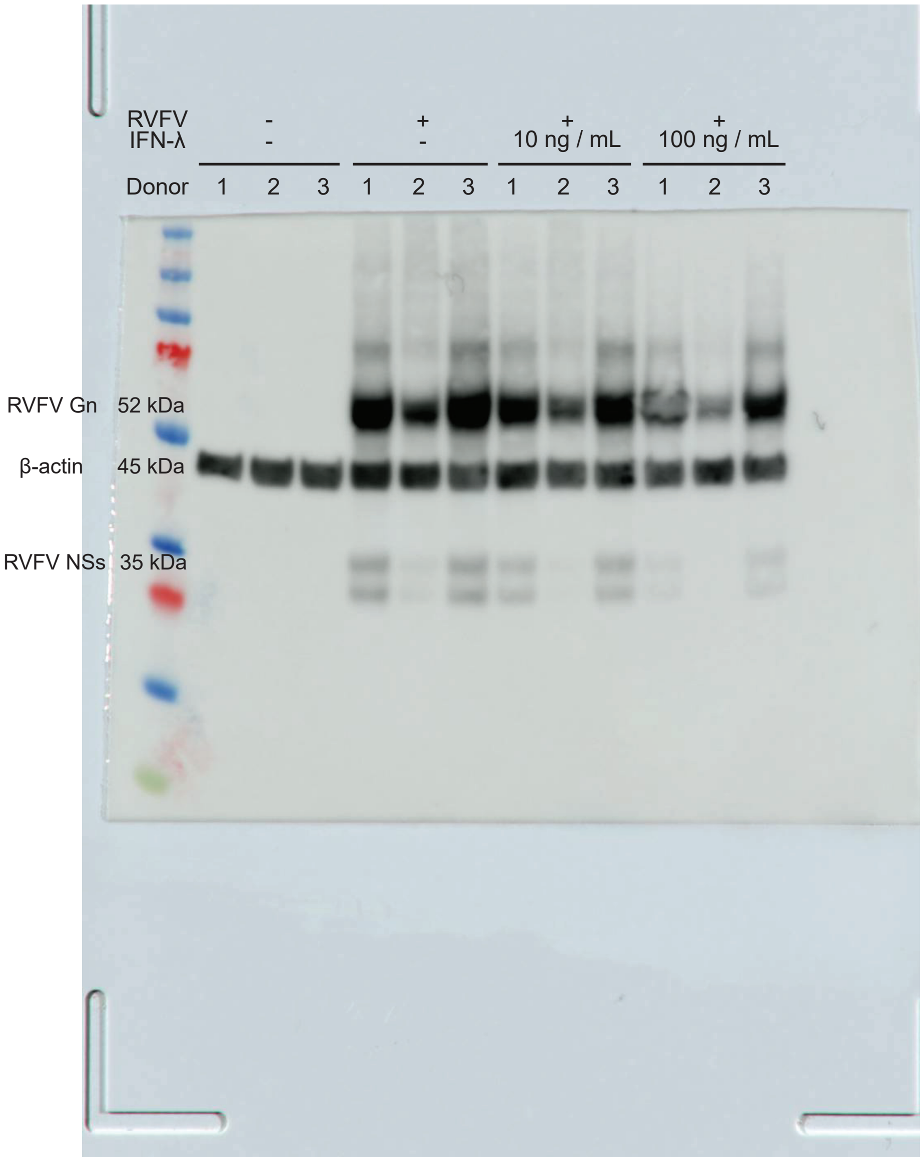

Original Western Blot
Figure 3 C, Experiment 3

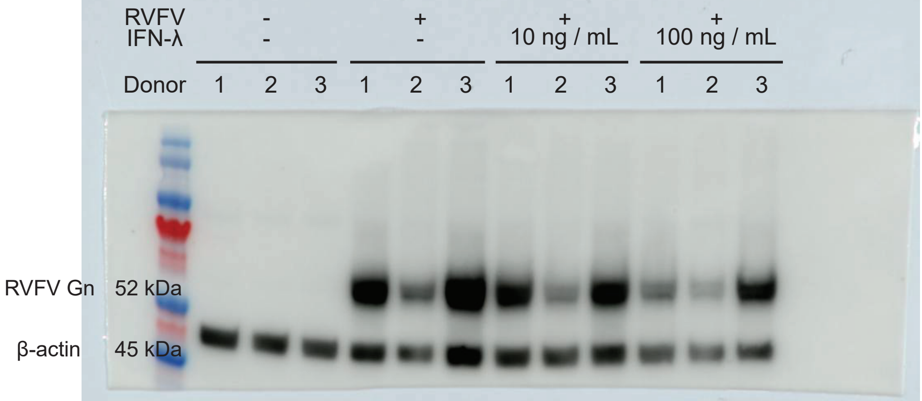

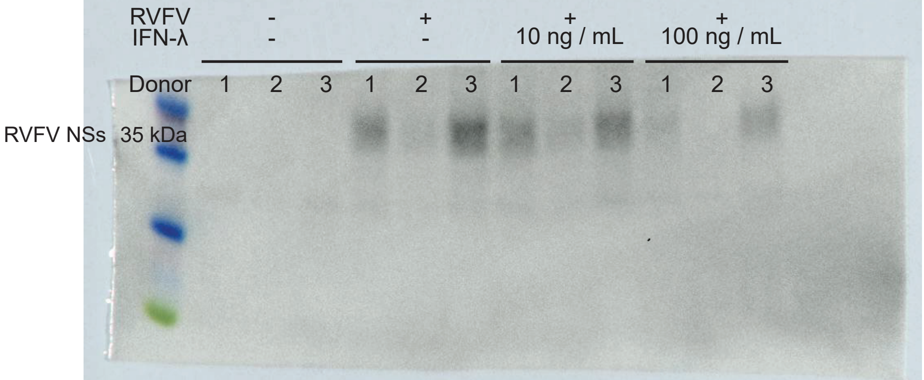

Supplement: Document S2. Original images _spl_Western blotting_spl_260327 [file mmc2.pdf]
